# Properties of mesons in a strong magnetic field

**DOI:** 10.1140/epjc/s10052-016-4123-8

**Published:** 2016-06-02

**Authors:** Rui Zhang, Wei-jie Fu, Yu-xin Liu

**Affiliations:** 1 Department of Physics, State Key Laboratory of Nuclear Physics and Technology, Peking University, Beijing, 100871 China; 2 Institut für Theoretische Physik, Universität Heidelberg, Philosophenweg 16, 69120 Heidelberg, Germany; 3 Collaborative Innovation Center of Quantum Matter, Beijing, 100871 China; 4 Center for High Energy Physics, Peking University, Beijing, 100871 China

## Abstract

By extending the $\Phi$-derivable approach in the Nambu–Jona-Lasinio model to a finite magnetic field we calculate the properties of pion, $\sigma$, and $\rho$ mesons in a magnetic field at finite temperature not only in the quark–antiquark bound state scheme but also in the pion–pion scattering resonant state scenario. Our calculation as a result makes manifest that the masses of $\pi ^{0}$ and $\sigma$ meson can be nearly degenerate at the pseudo-critical temperature which increases with increasing magnetic field strength, and the $\pi ^{\pm }$ mass ascends suddenly at almost the same critical temperature. Meanwhile the $\rho$ mesons’ masses decrease with the temperature but increase with the magnetic field strength. We also check the Gell-Mann–Oakes–Renner relation and find that the relation can be violated clearly with increasing temperature, and the effect of the magnetic field becomes pronounced around the critical temperature. With different criteria, we analyze the effect of the magnetic field on the chiral phase transition and find that the pseudo-critical temperature of the chiral phase cross, $T_\mathrm{c}^{\chi }$, is always enhanced by the magnetic field. Moreover, our calculations indicate that the $\rho$ mesons will get melted as the chiral symmetry has not yet been restored, but the $\sigma$ meson does not disassociate even at very high temperature. Particularly, it is the first to show that there does not exist a vector meson condensate in the QCD vacuum in the pion–pion scattering scheme.

## Introduction

The properties of strong interaction matter (QCD matter) have attracted great attention in the past years, and plenty of theoretical and experimental results were obtained (see, for example, Refs. [1–42]). The complicated phase structure of the matter provides rich information on the property of the strong interaction at finite temperature and/or density, and it may shed light on the fundamental understanding for some basic problems, e.g., the origin of most mass of visible matter and the evolution of the early universe matter. The chiral phase transition which is expected to occur in ultrarelativistic heavy-ion collisions [1–4, 35] and/or in the interior of the highly compact stars [43–45] is one of the significant issues for the research of QCD matter. An important method to extract the information of the chiral phase transition is analyzing the variation of the hadrons’ properties in the medium at finite temperature and/or density (chemical potential), even in a finite magnetic field, compared with those at zero temperature, zero chemical potential, and zero magnetic field strength [37, 46–59]. Among the hadrons, mesons are more important than baryons at present, because the former are more sensitive to the change of the surroundings and related to the chiral phase transition more directly [58, 60]. It is known that, when the early universe experienced the cosmological electro-weak phase transition, the strength of the magnetic field may reach up to $eB \approx 200m_{\pi }^{2}$ [61]. In heavy-ion collision experiments, the magnetic field produced at the early stage of non-central collisions can be of the order of $eB \approx 0.1 m_{\pi }^{2}$ for SPS, $eB \approx m_{\pi }^{2}$ for RHIC, and $eB \approx 15 m_{\pi }^{2}$ for LHC [62]. Even though it is weaker than that at the early stage of the universe, the magnetic field produced in RHIC and/or LHC has been strong enough to influence the strong interaction matter significantly [36]. The effects of the magnetic field on the masses of hadrons and weak decay constant of the neutral pion have then been investigated [59, 63–67]. Since they are significant to check the validity of the Vafa–Witten theorem for the QCD vacuum, the variation behaviors of the $\rho$ meson masses with respect to the magnetic field strength have also been studied [63–67]. However, the temperature and magnetic field strength dependence of the $\rho$ meson mass and the existence of the $\rho$ meson condensate in a very strong magnetic field at high temperature are still under debate (see, e.g., Refs. [63–65] and Refs. [66, 67]). In this paper, we consider further the 2 flavor QCD matter and the properties of mesons, including not only the pseudoscalar neutral pion and scalar $\sigma$ meson but also the charged pion and $\rho$ mesons, and we show the impossibility of the $\rho$ meson condensate by analyzing the variation behavior of the masses and the width of the mass pole.

In a general point of view, researches on the magnetic field effect have been carried out much more widely and much progress has been made, e.g., there may exist a chiral magnetic effect, which demonstrates that imbalanced chirality in a magnetic field can induce a current along the magnetic field and this results in a separation of electric charges [36, 41, 68–76]. Through the research simultaneously some open questions have arisen. One of them is whether there exists magnetic catalysis, which says that the quark condensate would be enhanced with increasing the magnetic field [77–81]. A direct consequence of magnetic catalysis is that the critical temperature of the chiral phase transition increases monotonically with the increasing of the magnetic field [28, 29, 79–84]. However, the latter lattice QCD calculations show an opposite behavior, called inverse magnetic catalysis, which presents a decreasing or non-monotonous behavior of the critical temperature with increasing magnetic field [30–32, 85]. Lots of works have been accomplished in order to determine whether the magnetic catalysis or the inverse catalysis is correct (see, for instance, Refs. [30–32, 86–101]), but it is still a puzzle (for a review, see Ref. [102]). Since mesons carry lots of information on the dynamical chiral symmetry breaking (DCSB) and restoration (in particular, pions), and the proposed enhancement of the meson or quark–antiquark pair condensate induced by the strong magnetic field [83, 84, 86] may be a signature of the magnetic catalysis, it is then expected that studies of meson properties in a strong magnetic field in this paper would shed light on this open question.

If we consider the charged mesons ($\pi ^\pm$ and $\rho ^\pm$) as point particles in an external magnetic field *B*, which is along the *z* direction, the energy level of the particle with mass *m* and spin *s* can be expressed as [37]

1 $$\begin{aligned} \varepsilon ^2_{n,s_z}(p_z)=p^2_z+(2n-2s_z+1)eB+m^2, \end{aligned}$$

where *n* denotes the order number of the Landau level. As mentioned in Ref. [37], for the pion $s_{z}=0$, and for the $\rho$ meson $s_{z}=1$; then the ground state mass of the charged pion and $\rho$ meson are given as

2 $$\begin{aligned}&m^2_{\pi ^\pm }(B)=m^2_{\pi ^\pm }+eB, \end{aligned}$$

3 $$\begin{aligned}&m^2_{\rho ^\pm }(B)=m^2_{\rho ^\pm }-eB, \end{aligned}$$

where $m^2_{\pi ^\pm }$ and $m^2_{\rho ^\pm }$ are the zero-field vacuum masses of the $\pi ^\pm$ and $\rho ^\pm$. However, mesons are not point particles. We cannot ignore the contribution of the internal quark structure of the particle to its mass, so in this paper we will calculate the mesons’ masses from the internal quark–antiquark contribution and at the same time make a correction on the point particle approximation.

It has been known that the Nambu–Jona-Lasinio (NJL) model is a QCD-inspired model [103–106], which demonstrates the effects of chiral symmetry and its breaking well and, in turn, can describe the meson properties at finite temperature successfully. Meanwhile, the $\Phi$-derivable approximation [107–110] has been known as a non-perturbative approach to the quantum field theory [111, 112], at least a two-particle-irreducible effective action formalism [113]. In this paper, we take the NJL model with an extension of the $\Phi$-derivable scheme to a finite magnetic field to calculate the meson properties in the conventional view that the mesons are quark and antiquark bound states. However, the $\sigma$ meson may not be a simple quark–antiquark bound state (see, e.g., Refs. [114–116]) but a resonant state of pion–pion scattering, and so does the $\rho$ meson (for reviews, see e.g., Refs. [117–121]). We will also study the mesons’ properties by analyzing the $\pi$–$\pi$ scattering lengths and the resonant states.

This paper is organized as follows. In Sect. 2, we briefly reiterate the scheme of describing the mesons in view of their internal quark–antiquark structure in the 2 flavor NJL model with the $\Phi$-derivable scheme at finite temperature but zero magnetic field. In Sect. 3, we extend the formulation to the case of a finite magnetic field. In Sect. 4, numerical results and discussions of the dependence of meson properties on the magnetic field are presented. In Sect. 5, we re-calculate the masses and the widths of the $\sigma$ and $\rho$ mesons in view of the $\pi$–$\pi$ scattering resonant states to reanalyze the effect of the magnetic field in an alternative scheme. In Sect. 6, we will give a summary and make some remarks.

## Meson properties in the NJL model without magnetic field

We begin with the NJL Lagrangian:

4 $$\begin{aligned} \mathcal {L}= & {} \overline{\psi }(i\partial \!\!\!/ - m_{0}) \psi + g_\mathrm{s} [(\overline{\psi }\psi )^{2} + (\overline{\psi } i \gamma _{5} \overrightarrow{\tau }\psi )^{2} ] \nonumber \\&- g_\mathrm{v} (\overline{\psi } \gamma ^{\mu } \overrightarrow{\tau } \psi )^{2} , \end{aligned}$$

where $\psi$ and $\overline{\psi }$ denote a quark and an antiquark field with $N_\mathrm{f}$ flavors and $N_\mathrm{c}$ colors, $m_{0}$ is the bare quark mass, $\tau ^{i}(i=1,2,3)$ are the Pauli matrices in flavor space. The effective four fermion interaction constant for scalar and pseudoscalar channels is $g_\mathrm{s}$, and that for the vector channel is $g_\mathrm{v}$. In this paper we always treat $N_\mathrm{f}$ and $N_\mathrm{c}$ as constants, $N_\mathrm{f}=2$ and $N_\mathrm{c}=3$.

To make use of the $\Phi$-derivable theory in practical calculations, we follow exactly the scheme described in Refs. [110, 122]. We skip the complicated derivations and only list some important results as follows. At first, the constituent quark mass can be derived from the gap equation,

5 $$\begin{aligned} M=m_{0} + \Sigma \, , \end{aligned}$$

where $\Sigma$ is the quark self-energy function. In the lowest order approximation, it reads

6 $$\begin{aligned} \Sigma = 2 {g_\mathrm{s}} \int \frac{\mathrm{d}^4q}{(2\pi )^4}\mathrm {tr}(iS(q)), \end{aligned}$$

with full quark propagator $S(q)=1/(q \!\!\!/ - M)$. The trace notation, tr, acts in the Dirac, flavor, and color spaces. The quark condensate $\langle \overline{q}q\rangle$ is defined as

7 $$\begin{aligned} \langle \bar{q} q \rangle = \phi =-\int \frac{\mathrm{d}^4q}{(2\pi )^4}\mathrm {tr}(iS(q)). \end{aligned}$$

To show the flavor dependence of the condensate, the trace in Eq. (7) does not include that in flavor space usually. Comparing with Eqs. (6) and (5) we get

8 $$\begin{aligned} \phi =-\frac{M - m_{0}}{4{g_\mathrm{s}}}. \end{aligned}$$

In view of the fact that mesons are bound states of a quark and an antiquark, the meson propagators can be represented in terms of its “polarization function” $\Pi _{\alpha }(p)$ as

9 $$\begin{aligned}&D_\sigma (p)=\frac{2g_\mathrm{s}}{1-2g_\mathrm{s}\Pi _\sigma (p)}, \end{aligned}$$

10 $$\begin{aligned}&D_\pi (p)=\frac{2g_\mathrm{s}}{1-2g_\mathrm{s}\Pi _\pi (p)}, \end{aligned}$$

11 $$\begin{aligned}&D_\rho (p)=\frac{2g_\mathrm{v}}{1-2g_\mathrm{v}\Pi _\rho (p)}. \end{aligned}$$

The “polarization functions” can be written as

12 $$\begin{aligned} \Pi _{\alpha } (p) = -i \int \frac{\mathrm{d}^{4}q}{(2\pi )^{4}} tr[iS(q+p) \Gamma _{\alpha } i S(q) \Gamma _{\alpha } ], \end{aligned}$$

where $\alpha = \sigma , \pi , \rho$ denotes the scalar, pseudoscalar, and vector channel, respectively. The $\Gamma _{\alpha }$ is correspondingly 1, $i\gamma _{5} \overrightarrow{\tau }$, and $\gamma ^{\mu } \overrightarrow{\tau }$ for the three channels. It is remarkable that, even though Eq. (12) is similar to that in the usual NJL model (see, e.g., Refs. [66, 103]), it is in fact the same as that in the Bethe–Salpeter equation when calculating the four quark interaction kernel [58, 122–124]. To show this we take the terminology of polarization function(s) with quotation marks in our context.

After some tedious calculations, one obtains the “polarization functions” as

13 $$\begin{aligned}&\Pi _\sigma (p)=4iN_\mathrm{c} N_\mathrm{f} \left[ I_{1} -\frac{1}{2} (p^{2} - 4M^{2}) I(p) \right] , \end{aligned}$$

14 $$\begin{aligned}&\Pi _\pi (p)=4iN_\mathrm{c} N_\mathrm{f} \left[ I_{1} - \frac{1}{2}p^{2} I(p) \right] , \end{aligned}$$

15 $$\begin{aligned}&\Pi _\rho (p)=-8i N_\mathrm{c} N_\mathrm{f} \left[ I_{1} - \frac{1}{2} (p^{2} + 2 M^{2}) I(p) \right] , \end{aligned}$$

where *M* is the constituent quark mass, and

16 $$\begin{aligned} I_{1}= & {} \int \frac{\mathrm{d}^4q}{(2\pi )^4}\frac{1}{q^2-M^2}\, , \end{aligned}$$

17 $$\begin{aligned} I(p)= & {} \int \frac{\mathrm{d}^4q}{(2\pi )^4}\frac{1}{ [ (q+p)^2-M^2 ] (q^2-M^2)}\, . \end{aligned}$$

From the pole of the meson propagators, we can obtain the meson mass from the equation

18 $$\begin{aligned} 1-2g_\alpha \Pi _\alpha (m_\alpha )=0 \, . \end{aligned}$$

The pion decay constant $f_\pi$ can be calculated from the vacuum to one-pion axial-vector matrix element. After some calculations we have the following form for $f_{\pi }$:

19 $$\begin{aligned} f_{\pi }^{2} = -4 i N_\mathrm{c} M^2 I(0), \end{aligned}$$

where *I*(0) is defined in Eq. (17), but with $p=0$.

So far, we have only given formulas for the case of zero temperature. To take into account the effect of the finite temperature, we adopt in this paper the Matsubara formalism. In this formalism, the energy part is replaced by the Matsubara frequencies $i\omega _{n}$ with $\omega _{n} = 2n{\pi }T$ for bosons and $\omega _{n} = (2n+1){\pi }T$ for fermions. Then the integral can be given as

20 $$\begin{aligned} \int \frac{\mathrm{d}^4p}{(2\pi )^4}f(p^0,\overrightarrow{p}) = i\beta ^{-1} \sum _n \int \frac{\mathrm{d}^3p}{(2\pi )^3}f(i\omega _{n},\overrightarrow{p}) \, , \end{aligned}$$

where $\beta =1/T$ is the inverse of the temperature. Then the gap equation can be rewritten by

21 $$\begin{aligned} M=m_{0} + 4{g_\mathrm{s}} N_\mathrm{c} N_\mathrm{f} \int \frac{\mathrm{d}^3q}{(2\pi )^3}\frac{M}{E_{q}} [ 1 - 2n_{f}(E_{q}) ] \, , \end{aligned}$$

with

22 $$\begin{aligned}&E_{q} = \sqrt{q^2 + M^2}\, , \end{aligned}$$

23 $$\begin{aligned}&n_{f} (E_{q}) = \frac{1}{e^{{\beta }E_{q}} + 1} \, . \end{aligned}$$

For the “polarization functions”, the integrals are

24 $$\begin{aligned} I_{1} = -i\int \frac{\mathrm{d}^3q}{(2\pi )^3}\frac{1}{2E_{q}}(1-2 n_{f}(E_{q})) \, , \end{aligned}$$

25 $$\begin{aligned} I(p)= & {} i\int \frac{\mathrm{d}^3q}{(2\pi )^3}\frac{1}{4 E_{q} E_{q+p}} \left[ \phantom {\left( \frac{1}{p_{0} + E_{q} + E_{q+p}} - \frac{1}{p_{0} - E_{q} - E_{q+p}}\right) } \left[ n_{f} (E_{q} ) - n_{f} (E_{q+p} ) \right] \right. \nonumber \\&\times \left( \frac{1}{p_{0} + E_{q} - E_{q+p} } - \frac{1}{p_{0} - E_{q} + E_{q+p} }\right) \nonumber \\&+ \left[ 1 - n_{f} (E_{q} ) - n_{f} (E_{q+p} ) \right] \nonumber \\&\left. \times \left( \frac{1}{p_{0} + E_{q} + E_{q+p} } - \frac{1}{p_{0} - E_{q} - E_{q+p} }\right) \right] .\nonumber \\ \end{aligned}$$

For the simple case that the external three-momentum $\vec {p}=0$, *I*(*p*) has a more simple form,

26 $$\begin{aligned} I(p) = i\int \frac{\mathrm{d}^3q}{(2\pi )^3} [ 1 - 2 n_{f} (E_{q} ) ] \left( \frac{1}{p_{0} + 2E_{q} } -\frac{1}{p_{0} - 2E_{q} } \right) . \nonumber \\ \end{aligned}$$

With Eqs. (24) and (26) we can solve Eq. (18) to obtain the mesons’ masses. One can notice easily then

27 $$\begin{aligned} (m_{\sigma }^{2} - 4 M^{2} ) I(m_{\sigma } ) = m_{\pi }^{2} I (m_{\pi } ) \, . \end{aligned}$$

Since the function *I*(*p*) is usually a very smooth function of *p* and depends on *p* quite weakly [103], one can have the approximation $I(m_{\sigma } ) = I(m_{\pi } ) = I(0)$. As a consequence, one has

28 $$\begin{aligned} m_{\sigma }^{2} = m_{\pi }^{2} + 4 M^{2} \, . \end{aligned}$$

It is apparent, as there exactly exist a chiral symmetry, $M = 0$, that $m_{\sigma } = m_{\pi }$. Therefore the degeneracy of the $\sigma$ meson and pion masses is usually regarded as a signal of the chiral symmetry restoration.

## In an external magnetic field

With an external magnetic field, the NJL Lagrangian is given as

29 $$\begin{aligned} \mathcal {L}= & {} \overline{\psi }(i\partial \!\!\!/ - q_{f} eA \!\!\!/ - m_{0} )\psi +{g_\mathrm{s} } [(\overline{\psi }\psi )^2+(\overline{\psi }i\gamma _{5} \overrightarrow{\tau }\psi )^2] \nonumber \\&-{g_\mathrm{v} } (\overline{\psi }\gamma ^\mu \overrightarrow{\tau } \psi )^2\, , \end{aligned}$$

where $$q_{f} $$ is the quark electric charge number, 2 / 3 for up quark and $$-1/3$$ for down quark. We assume a homogeneous external magnetic field *B* along the *z*-direction, then *A* can be chosen as

30 $$\begin{aligned} A=\left( 0,-\frac{1}{2}Bx_2,\frac{1}{2}Bx_1,0\right) . \end{aligned}$$

The quark propagator has the form

31 $$\begin{aligned} S(q)=\frac{1}{q \!\!\!/ - q_{f} eA \!\!\!/ - M} =\frac{q \!\!\!/ - q_{f} eA \!\!\!/ + M}{(q - q_{f} eA)^{2} - M^{2} } \, . \end{aligned}$$

After some calculations, part of the denominator of the above equation can be expressed as

32 $$\begin{aligned} (q - q_{f} eA)^2 = q_{0}^{2} - (q_{3}^{2} + 2n|q_{f} |eB) \, , \end{aligned}$$

which means that, because of the existence of the external magnetic field, the transverse part of the three-momentum which is perpendicular to the *z*-direction is quantized as discrete Landau levels. Then the $\Phi$-derivable scheme and all the equations in the previous section can easily be extended to finite magnetic field. We list the main ones in the following.

The gap equation is given as

33 $$\begin{aligned} M = m_{0} + 2{g_\mathrm{s} } N_\mathrm{c} \sum _{f} \! \frac{|q_{f} |eB}{2\pi }\! \sum _{n=0}^\infty \! \alpha _{n} \!\! \int \!\! \frac{\mathrm{d}q_{3} }{2\pi }\frac{M}{E_{q} } [1 - 2 n_{f} (E_{q} ) ] \, , \end{aligned}$$

with

34 $$\begin{aligned} E_{q}^{2} = q_{3}^{2} + 2n|q_{f} | eB + M^{2} \, , \end{aligned}$$

and $\alpha _{n}$ is the spin degeneracy factor,

35 $$\begin{aligned} \alpha _{n} = {\left\{ \begin{array}{ll} 1&{}\quad {n=0},\\ 2&{}\quad \text {otherwise}. \end{array}\right. } \end{aligned}$$

The “polarization function” of the $\sigma$ meson in a magnetic field is given by

36 $$\begin{aligned} \Pi _{\sigma }(p) = 2 i N_\mathrm{c} \sum _{f} \frac{|q_{f} |eB}{2\pi } \left[ I_{1} - \frac{1}{2}(p^{2} - 4M^{2})I(p) \right] \, , \end{aligned}$$

with

37 $$\begin{aligned} I_{1}= & {} - i \sum _{n} \alpha _{n} \int \frac{\mathrm{d}{q_{3} }}{2\pi }\frac{1}{2E_{q} } [1-2n_{f} (E_{q} ) ] \, , \end{aligned}$$

38 $$\begin{aligned} I(p)= & {} i \sum _{n} \alpha _{n} \int \frac{\mathrm{d}{q_{3} }}{2\pi }\frac{1}{4 E_{q} E_{q+p} } \left[ [n_{f} (E_{q} ) - n_{f} (E_{q+p} ) ] \right. \nonumber \\&\left. \times \left( \frac{1}{p_{0} + E_{q} - E_{q+p} } -\frac{1}{p_{0} - E_{q} + E_{q+p} } \right) \right. \nonumber \\&\left. +\, [ 1 - n_{f} (E_{q} ) - n_{f} (E_{q+p} ) ] \right. \nonumber \\&\left. \times \left( \frac{1}{p_{0} + E_{q} +E_{q+p} } - \frac{1}{p_{0} - E_{q} - E_{q+p} }\right) \right] .\nonumber \\ \end{aligned}$$

Considering the zero three-momentum, $\vec {p}=0$, the “polarization function” can be simply written as

39 $$\begin{aligned} \Pi _{\sigma }(p_{0} )= & {} \frac{M - m_{0} }{2 {g_\mathrm{s} } M} + 2 N_\mathrm{c} \sum _{f} \frac{|q_{f} |eB}{2\pi }\frac{1}{2}(p_{0}^{2} - 4 M^2)\sum _{n} \alpha _{n} \nonumber \\&\times \! \int \! \frac{\mathrm{d}{q_{3} }}{2\pi }\frac{1}{4E_{q}^{2}} [1 - 2 n_{f} (E_{q} ) ] \left( \frac{1}{p_{0} - 2E_{q} } - \frac{1}{p_{0} + 2 E_{q} } \right) .\nonumber \\ \end{aligned}$$

For the pion, we should notice that the form of the “polarization function” in the case of zero magnetic field includes the contributions of quark–antiquark loops for both *u* ($$\bar{u}$$) and *d* ($$\bar{d}$$) quarks, thus the mass we get is actually that of the neutral pion $$\pi ^{0}$$. It is also obvious that without a magnetic field we cannot distinguish the charged pion $$\pi ^{\pm }$$ from the neutral $$\pi ^{0}$$ in view of the “polarization functions”. In fact, when determining the parameters, one usually fixes the pion mass as the $$\pi ^{0}$$ mass. When considering a finite magnetic field, everything changes. The isospin Pauli matrix for the neutral $$\pi ^{0}$$ takes $$\tau ^{3}$$, and for the charged $$\pi ^{\pm }$$ takes $$\tau ^{\pm } =(\tau ^{1}{\pm } i\tau ^{2})/\sqrt{2}$$. For the neutral $$\pi ^{0}$$ we can directly get the “polarization function”, which has almost the same form as Eq. (14) except for the three-momentum integrations being replaced by a sum of the Landau level and an integration of $$p_{3} $$,

40 $$\begin{aligned} \Pi _{\pi ^{0}}(p) = 2 i N_\mathrm{c} \sum _{f} \frac{|q_{f} |eB}{2\pi } \left[ I_{1} -\frac{1}{2}p^{2} I(p) \right] \, , \end{aligned}$$

where $I_{1}$ and *I*(*p*) are the same as Eqs. (37) and (38). With $\vec {p}=0$,

41 $$\begin{aligned} \Pi _{\pi ^{0}}(p_{0} )= & {} \frac{M - m_{0} }{2{g_\mathrm{s} }M} + 2 N_\mathrm{c} \sum _{f} \frac{|q_{f} |eB}{2\pi }\frac{1}{2}p_{0}^{2} \sum _{n} \alpha _{n} \nonumber \\&\times \int \! \frac{\mathrm{d}{q_{3} }}{2\pi }\frac{1}{4E_{q}^{2}} [1 \! - \! 2 n_{f} (E_{q} ) ]\nonumber \\&\times \left( \frac{1}{p_{0} \! - \! 2E_{q} } - \frac{1}{p_{0} \! + \! 2 E_{q} } \right) \, . \end{aligned}$$

For the charged $\pi ^{\pm }$, we can start from the inner structure of $\pi ^{\pm }$. Different from $\pi ^{0}$, $\pi ^{+}$ is composed of *u* and $\overline{d}$ quark, so that the quark loop structure of summing over the *u* ($\bar{u}$) and *d* ($\bar{d}$) quarks for $\pi ^{0}$ should be replaced by one *u* quark and one $\overline{d}$ quark for $\pi ^{+}$. Similarly for $\pi ^{-}$. Thus the “polarization function” has the form (here we also consider the case of zero three-momentum)

42 $$\begin{aligned} \Pi _{\pi ^{\pm }}(p) = 2 i N_\mathrm{c} \frac{eB}{2\pi } \left[ I_{1} - \frac{1}{2} p^{2} I(p) \right] , \end{aligned}$$

with

43 $$\begin{aligned} I(p)= & {} i\sum _{n} \alpha _{n} \int \frac{\mathrm{d}q_3}{2\pi }\frac{1}{4E_{q} E_{q}^{\prime }} \left[ [ n_{f} (E_{q} ) - n_{f} (E_{q}^{\prime }) ] \right. \nonumber \\&\left. \times \left( \frac{1}{p_{0} + E_{q} - E_{q}^{\prime }} - \frac{1}{p_{0} - E_{q} + E_{q}^{\prime }} \right) \right. \nonumber \\&\left. + [ 1 - n_{f} (E_{q} ) - n_{f} (E_{q}^{\prime }) ] \right. \nonumber \\&\left. \times \left( \frac{1}{p_{0} + E_{q} + E_{q}^{\prime }} - \frac{1}{p_{0} - E_{q} - E_{q}^{\prime }}\right) \right] ,\end{aligned}$$

44 $$\begin{aligned} E_{q}^{2}= & {} 2n|q_{u} |eB + q_{3}^{2} + M^{2},\end{aligned}$$

45 $$\begin{aligned} {E_{q}^{\prime }}^{2}= & {} 2 n^{\prime }|q_{d} |eB + q_{3}^{2} + M^{2}. \end{aligned}$$

Due to the constraints of conservation of momentum, *n* and $n^{\prime }$ have the relation $n^{\prime }=2n$. Then Eq. (43) can be reduced to the simple form

46 $$\begin{aligned} I(p)= & {} i \sum _{n} \alpha _{n} \!\! \int \!\! \frac{\mathrm{d}{q_{3} }}{2\pi } [ 1 - 2 n_{f} (E_{q} ) ] \nonumber \\&\times \left( \frac{1}{p_{0} \! + \! 2 E_{q} } - \frac{1}{p_{0} \! - \! 2 E_{q} } \right) \, , \end{aligned}$$

with

47 $$\begin{aligned} E_{q}^{2} =\frac{4}{3} n e B + q_{3}^{2} + M^{2}\, . \end{aligned}$$

Similar to the pion, we can easily get the neutral $\rho ^{0}$ and charged $\rho ^{\pm }$ “polarization functions”

48 $$\begin{aligned} \Pi _{\rho ^{0}}(p_{0} )= & {} \frac{M-m_{0} }{{g_\mathrm{s} }M} \!+\!4N_\mathrm{c} \sum _{f} \frac{|q_{f} |eB}{2\pi }\frac{1}{2}(p_{0}^{2}\!+\! 2M^{2})\sum _{n}\alpha _{n} \nonumber \\&\times \!\! \int \!\! \frac{\mathrm{d}{q_{3} }}{2\pi }\frac{1}{4E_{q}^{2}} [1-2 n_{f} (E_{q} )]\nonumber \\&\times \left( \frac{1}{p_{0} \! - \! 2E_{q} } -\frac{1}{p_{0} \! + \! 2E_{q} } \right) \, , \end{aligned}$$

with the $$E_{q} $$ expressed in Eq. (34);

49 $$\begin{aligned} \Pi _{\rho ^{\pm }}(p_{0} )= & {} \frac{M-m_{0} }{{g_\mathrm{s} }M}+4N_\mathrm{c} \frac{eB}{2\pi }\frac{1}{2}(p_{0}^{2} +2M^{2})\sum _{n}\alpha _{n} \nonumber \\&\times \! \! \int \!\! \frac{\mathrm{d}{q_{3} }}{2\pi }\frac{1}{4E_{q}^{2}} [ 1 \! - \! 2 n_{f} (E_{q} ) ]\nonumber \\&\times \left( \frac{1}{p_{0} \! - \! 2 E_{q} } - \frac{1}{p_{0} \! + \! 2 E_{q} } \right) \, , \end{aligned}$$

with the $$E_{q} $$ expressed in Eq. (47).

Together with Eq. (18) we can get the corresponding meson mass.

The magnetic field strength dependence of the quark condensate at finite temperature can still be determined by Eq. (8) with the corresponding quark mass *M*. The pion decay constant can be given by

50 $$\begin{aligned} f_{\pi }^{2} = -i N_\mathrm{c} M^{2} \sum _{f} \frac{|q_{f} |eB}{2\pi } I(0), \end{aligned}$$

where *I*(0) is determined by Eq. (38) with $p=0$.

**Fig. 1:**
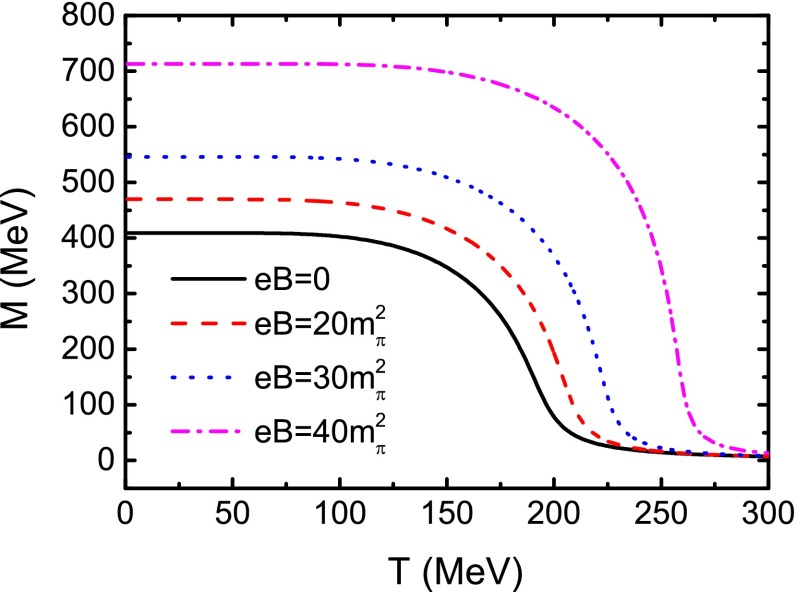
Calculated variation behavior of the constituent quark mass as a function of temperature at several values of magnetic field strength

## Numerical results and discussions

The feature of four fermion contact interactions of the NJL model makes the model nonrenormalizable, and an effective three-momentum cutoff $$\Lambda $$ is thus needed to regulate the divergent quantities. Together with the small bare quark mass $$m_{0} $$, the scalar interaction constant $$g_\mathrm{s} $$, and the vector interaction constant $$g_\mathrm{v} $$, there are four parameters in the NJL model. The parameters $$\Lambda $$, $$m_{0} $$, and $$g_\mathrm{s} $$ are usually taken as [106] $$\Lambda =587.9\;$$MeV, $$m_{0} =5.6\;$$MeV, $${g_\mathrm{s} }\Lambda ^{2} = 2.44$$, which are fixed by fitting the quantities at zero temperature: $$f_{\pi } =92.4\;$$MeV, $$m_{\pi } =135.0\;$$MeV, and $$\langle \overline{u}u\rangle ^{1/3}=-240.8\;$$MeV. The last parameter $$g_\mathrm{v} $$ is fixed as $$g_\mathrm{v}=1.39\times 10^{-6}\, \text {MeV}^{-2}$$ by fitting the zero temperature $$\rho $$ meson mass $$m_{\rho } =770.0\;$$MeV.

In the case of a strong magnetic field, the sharp three-momentum cutoff $\theta (\Lambda - |\vec {p}|)$ suffers from a cutoff artifact since the continuum momentum is replaced by the discrete Landau quantized one. To avoid this problem, a smooth cutoff ${f_{\Lambda } }(\vec {p})$ is introduced [70] by

51 $$\begin{aligned} {f_{\Lambda } }(\vec {p}) = \sqrt{\frac{\Lambda ^{2N}}{\Lambda ^{2N} + |\vec {p}|^{2N}}} \, . \end{aligned}$$

It is apparent that, in the limit of $N \rightarrow \infty$, ${f_{\Lambda } }(\vec {p})$ is reduced to the sharp cutoff form. In our practical calculation, we take Eq. (51) with $N=10$ for the cutoff parameter and the commonly used values listed above for the other three parameters.

**Fig. 2:**
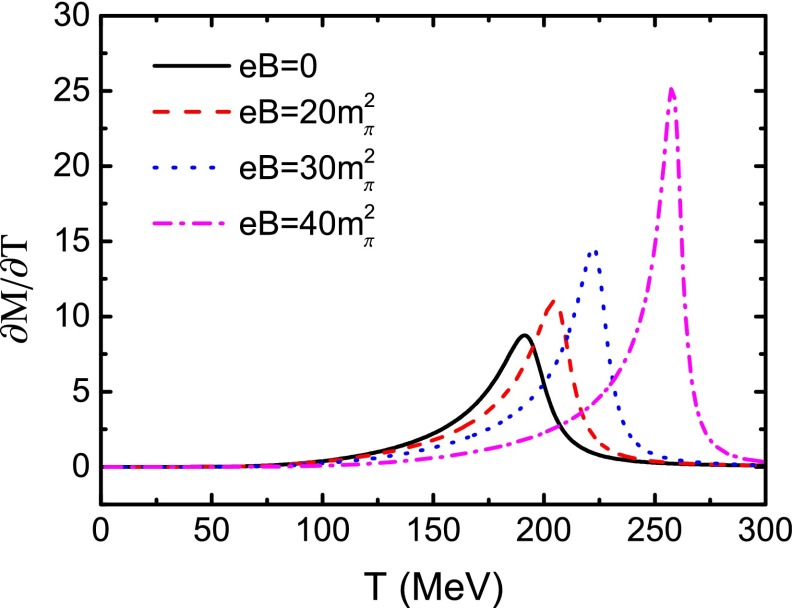
Calculated chiral susceptibility ${\partial }M/{\partial }T$ as a function of *T*, corresponding to the same values of *eB* in Fig. 1, respectively

In Fig. 1 we plot the calculated variation behavior of the constituent quark mass as a function of temperature with several values of the magnetic field strength. One can see that the constituent quark mass decreases quickly around a certain temperature for all the values of the magnetic field strength. We also plot the generalized chiral susceptibility ${\partial }M/{\partial }T$ as a function of temperature in Fig. 2. From the position of the peak of ${\partial }M/{\partial }T$, we can obtain, as usual, the chiral phase transition temperature for different magnetic field strengths, e.g., $T_\mathrm{c}=191\;$MeV at $eB=0$. When there is a finite magnetic field, Fig. 1 shows that the constituent quark mass increases with the magnetic field, and the strength of the phase transition increases with the magnetic field strength as well, as shown explicitly in Fig. 2, where both the height of the peaks and the (pseudo-)critical temperature increase with *eB*.

**Fig. 3:**
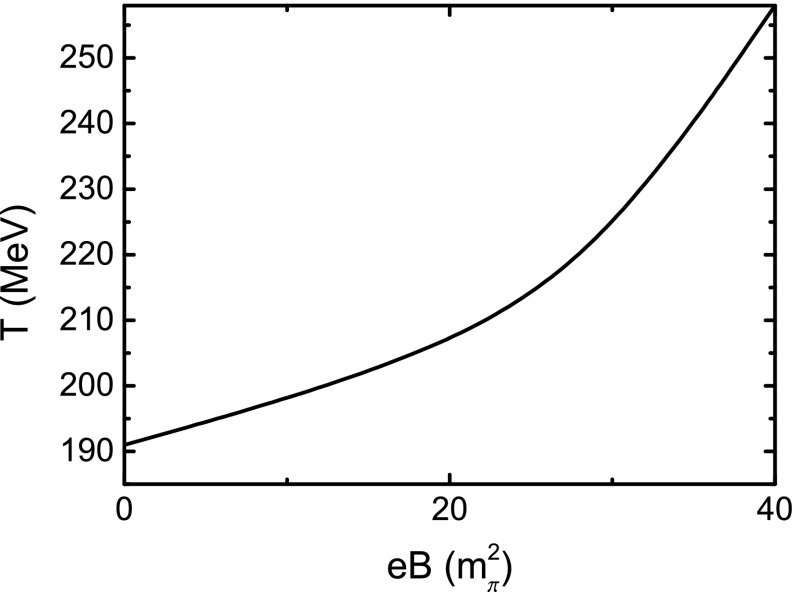
Calculated phase diagram in the *T*–*eB* plane

**Fig. 4:**
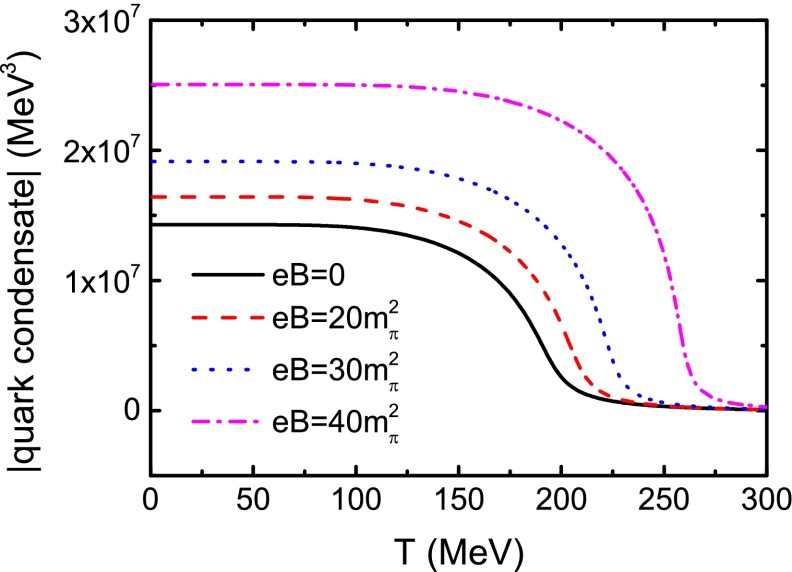
Calculated absolute value of the quark condensate as a function of the temperature at the same values of *eB* as those in Fig. 1

We show the critical temperature, or the pseudo-critical temperature more exactly, as a function of *eB* in Fig. 3 and list some of the values in Table 1. One can find from the figure and the table that the critical temperature increases with *eB*, in particular for a large magnetic field. This result implies a confirmation of the magnetic catalysis in the $\Phi$-derivable scheme with the NJL model. Figure 3 can also be treated as a phase diagram in the *T*–*eB* plane, where the regions above and below the curve correspond to the chiral symmetric phase and the DCSB phase, respectively. We also plot the absolute value of the quark condensate as a function of temperature in Fig. 4. Because of Eq. (8), we can get the same phase diagram as Fig. 3 via the criterion of $\partial \phi /{\partial }T$. In addition, the phase boundary in the *T*–*eB* plane can be parameterized as

52 $$\begin{aligned} T_\mathrm{c} = 191 + 1.827 (eB) - 0.109 (eB)^{2} + 0.00264 (eB)^{3} \, , \end{aligned}$$

with *eB* in unit $m_{\pi }^{2}$.

**Table 1:** Calculated pseudo-critical temperature $T_\mathrm{c}$ ’s in the case without and with external magnetic field, obtained with different criteria and the melting temperature of the $\rho$ meson, where the $T_\mathrm{c}^{\chi }$ stand for those with the constituent quark mass *M* and the chiral quark condensate $\langle \bar{q} q \rangle$, $T_\mathrm{c}^{{\pi }^{0}}$ for the $\pi ^{0}$ and $\sigma$ meson masses to begin to degenerate or nearly degenerate, $T_\mathrm{c}^{f_{\pi } }$ for the maximal decreasing rate of the $f_{\pi }$, $T_\mathrm{c}^{r}$ for the first minimum of the *r*, $T_\mathrm{m}^{{\rho }^{0}}$ for the $m_{{\rho }^{0}}$ to degenerate with the 2*M*, and $T_\mathrm{m}^{{\rho }^{\pm }}$ the highest for the mass solution to exist (all the temperatures are in unit MeV and the *eB* in $m_{\pi }^{2}$ at zero temperature and zero magnetic field)

*eB*	$T_\mathrm{c}^{\chi }$	$T_\mathrm{c}^{{\pi }^{0}}$	$T_\mathrm{c}^{f_{\pi } }$	$T_\mathrm{c}^{r}$	$T_m^{{\rho }^{0}}$	$T_m^{{\rho }^{\pm }}$
0.0	191	233	191	200	155	
10.0	201	256	201	211	194	169
20.0	205	277	205	214	215	173
30.0	222	304	222	232	245	196
40.0	258		258			

**Fig. 5:**
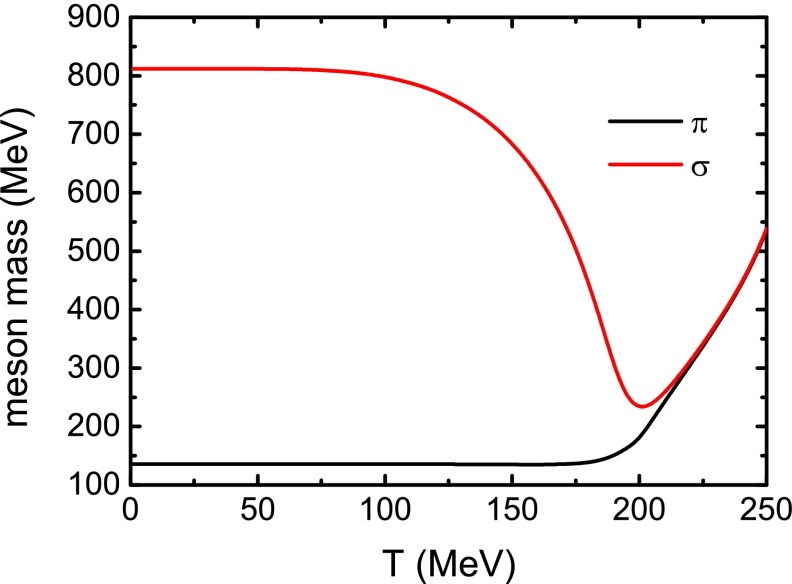
Calculated $\pi$ and $\sigma$ meson masses as functions of temperature *T* in the case of a vanishing magnetic field

The calculated $\pi$ and $\sigma$ meson masses as functions of temperature in the case of zero magnetic field are plotted in Fig. 5. Considering together with the variation behavior of the constituent quark mass, one can notice that the relation $m_{\sigma }^{2} = 4 M^{2} + m_{\pi }^{2}$ is conserved precisely. The degeneracy of the $\pi$ and $\sigma$ meson masses at high temperature implies evidently the restoration of the chiral symmetry.

Now we focus on the $\pi$ mass in the case of finite magnetic field. For the charged $\pi ^\pm$, we need to consider the contribution not only from the internal constituent quark and antiquark, but also from the point particle correction. In Eq. (2), the point particle correction is given for the case of zero temperature. We can directly extend it to the finite temperature case:

53 $$\begin{aligned} M^{2}_{\pi ^{\pm }}(T,eB) = m^{2}_{\pi ^{\pm }}(T,eB) + eB \, . \end{aligned}$$

In the above expression, $m_{\pi ^{\pm }}(T,eB)$ denotes the pion mass calculated from the constituent quark and antiquark contribution, where we can make the simplification of zero external momentum. This means that we consider the internal contribution to the meson mass in a static meson coordinate system. When considering the pion as a point particle moving in the external magnetic field, the momentum of the pion perpendicular to the direction of the magnetic field is quantized as the Landau levels, and the lowest Landau level governs the ground state of the pion mass, so that the point particle correction is a kinetic effect. The calculated masses of $\pi ^{0}$ and $\pi ^{\pm }$ in the case of a very weak magnetic field and zero temperature are 135.1, 142.4 MeV, respectively. Comparing with the experimental data $m_{\pi ^{+}} = 139.6$ MeV, one sees that the theoretical result of the $\pi ^\pm$ mass agrees with experiment very well (the error is only about $2\,\%$).

**Fig. 6:**
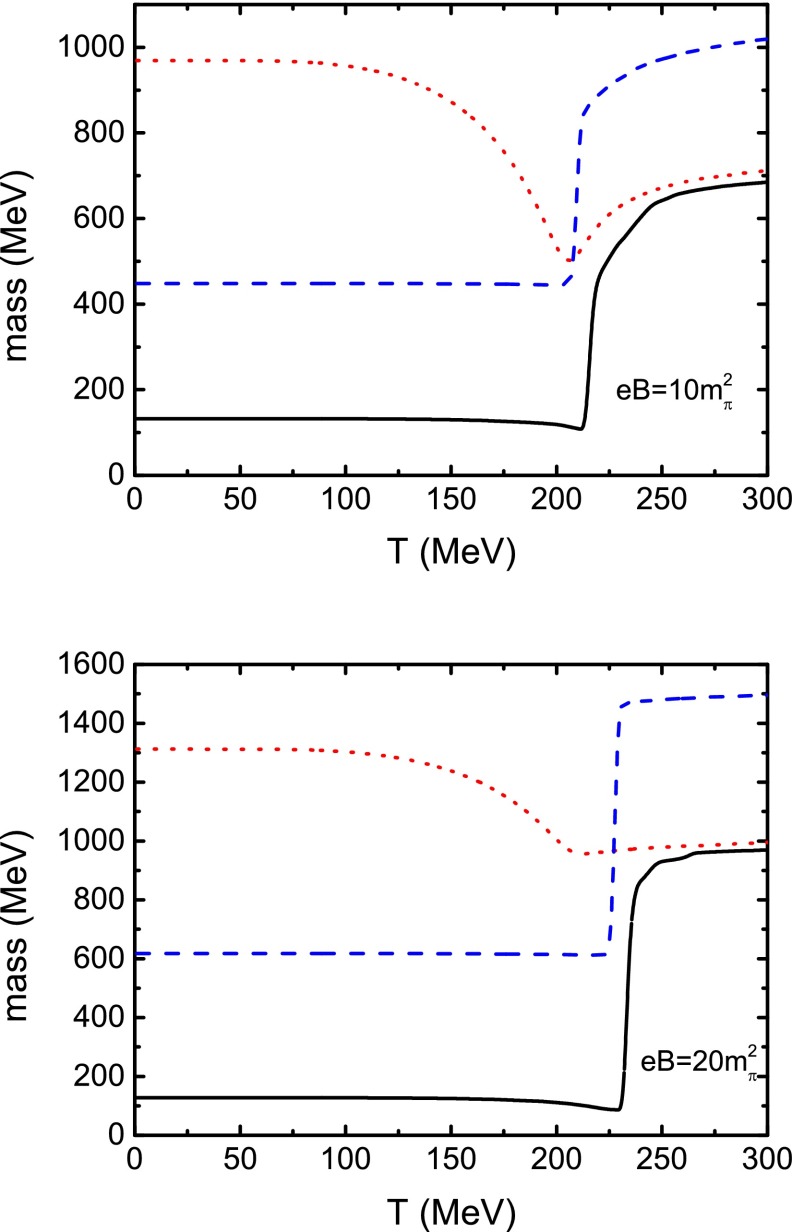
Calculated $\pi ^0$ and $\pi ^\pm$ masses (in *black solid*, *blue dashed line*, respectively) together with $\sigma$ meson mass (in *red dotted*) as functions of temperature at two values of the magnetic field strength

In Fig. 6 we illustrate the calculated masses of the $\pi ^{0}$ and $\pi ^{\pm }$ mesons together with that of the $\sigma$ meson as functions of temperature in two cases of nonzero magnetic field strength. The $\sigma$ meson mass, at a fixed temperature, shows a monotonic increasing behavior with the magnetic field strength. We can also find that in a weak magnetic field the $\sigma$ meson mass keeps the same behavior as that in the case of zero magnetic field, but with increasing magnetic field strength the temperature dependence of the $\sigma$ meson mass becomes weaker, especially at the temperature around the (pseudo-)critical one. This feature indicates that the $\sigma$ meson mass depends on the magnetic field more drastically than on the temperature. For the pions, it shows that there is almost no qualitative difference between the dependence of $\pi ^{0}$ and $\pi ^{\pm }$ masses on the temperature. When the temperature is lower than 191 MeV, which is the (pseudo-)critical temperature of the chiral phase transition without magnetic field, $T_\mathrm{c}^{\chi }$, the $\pi ^{0}$ mass is almost a constant. Once the temperature gets higher than the critical value, the behavior becomes a little complicated. The $\pi ^{0}$ mass decreases slightly around $T_\mathrm{c}^{\chi }$, and then it increases suddenly so as to become nearly degenerate with the $\sigma$ mass when the temperature is higher than the critical value $T_\mathrm{c}^{\chi }$. Different from the zero magnetic field case, the degeneration is not so precise. The temperatures for the mass difference ($m_{\pi ^{0}} - m_{\sigma }$) to be about $2\,\%$ of the $m_{\pi ^{0}}$ are listed in Table 1. The reason for the degeneracy to be not exact is the following. The existence of the magnetic field enhances the quark condensate and the constituent quark mass, but the temperature makes them decrease and the constituent quark mass will drop to that in dynamical chiral symmetry at higher temperature. From the relation between the masses of the $\sigma$ meson and the pion in Eq. (28), it is obvious that the degeneracy occurs at higher temperature. Another aspect is the finite current quark mass. When the temperature is above the critical one, the quark mass returns, in fact, to the current quark mass but not to zero. Therefore the pion mass does not equate the $\sigma$ mass precisely. From Fig. 6 we can also find that the critical temperature extracted from the ${\pi }^{0}$ mass increases with the magnetic field strength, which gives us similar information of the phase diagram in *T*–*eB* plane as shown in Fig. 3. However, there is a difference between the critical temperatures in the two cases, which implies that the phase transition is not a sharp (low order) phase transition, but a crossover. Moreover, the $\pi ^{\pm }$ mass increases with the magnetic field, no matter if the temperature is lower or higher than the critical value. The critical temperature for the $\pi ^{\pm }$ mass to increase abruptly is almost the same as that for the $\pi ^{0}$ meson (only about $6\;$MeV lower).

**Fig. 7:**
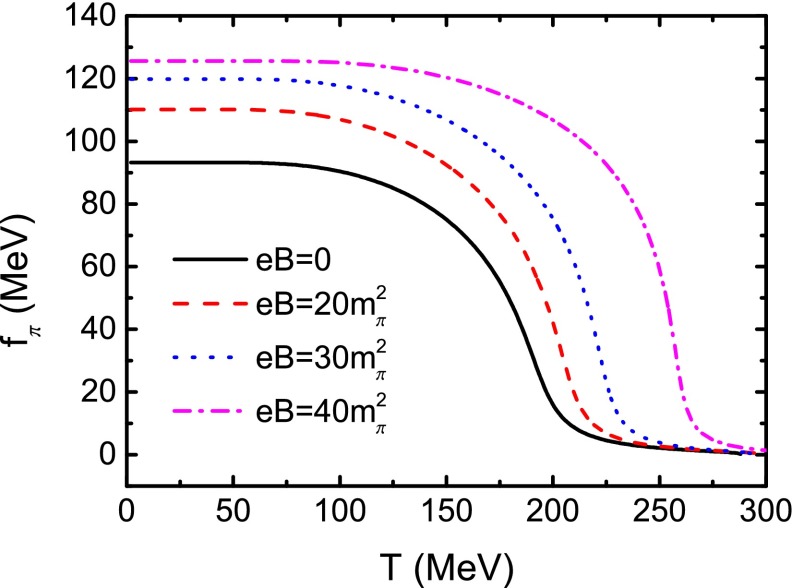
Calculated pion decay constant $f_{\pi }$ as a function of temperature at several values of magnetic field strength

It is well known that the Gell-Mann–Oakes–Renner (GOR) relation, which connects the $\pi$ mass and decay constant with the current quark mass and quark condensate, is a direct demonstration of the DCSB. The GOR relation reads

54 $$\begin{aligned} f_{\pi }^{2} m_{\pi }^{2} = - 2 m_{0} \langle \overline{q}q\rangle , \end{aligned}$$

where $2 \langle \overline{q}q\rangle$ includes the contributions of both the *u* and the *d* quarks. From Eqs. (19) and (50) we can get the $\pi$ decay constant. The obtained results at several values of the magnetic field strength are displayed in Fig. 7. We should note that the $\pi$ decay constant in Eq. (19) is related to the neutral pion $\pi ^0$, so we only consider the decay constant for $\pi ^0$ as shown in Eq. (50), even though the $\pi ^0$ and $\pi ^{\pm }$ can be distinguished in a magnetic field. From Fig. 7 we can notice that $f_{\pi }$ at a certain temperature increases with the magnetic field strength; and at a fixed magnetic field, $f_{\pi }$ decreases monotonously with the increasing of temperature and falls to zero at high temperature, which is just qualitatively the same as that given in Ref. [59]. Comparing the variation behavior with those of the constituent quark mass and the quark condensate, one can find that the critical temperature at which the decreasing rate of the $f_{\pi }$ takes its maximal value is exactly the same as that given with the constituent quark mass criterion (see Table 1). It is easy to check that the GOR relation is preserved very well at zero temperature and vanishing magnetic field. To examine the relation in the case of finite magnetic field, following Ref. [54] we define the ratio

55 $$\begin{aligned} r=\frac{f_\pi ^2m_\pi ^2}{- 2 m_0\langle \overline{q}q\rangle }. \end{aligned}$$

**Fig. 8:**
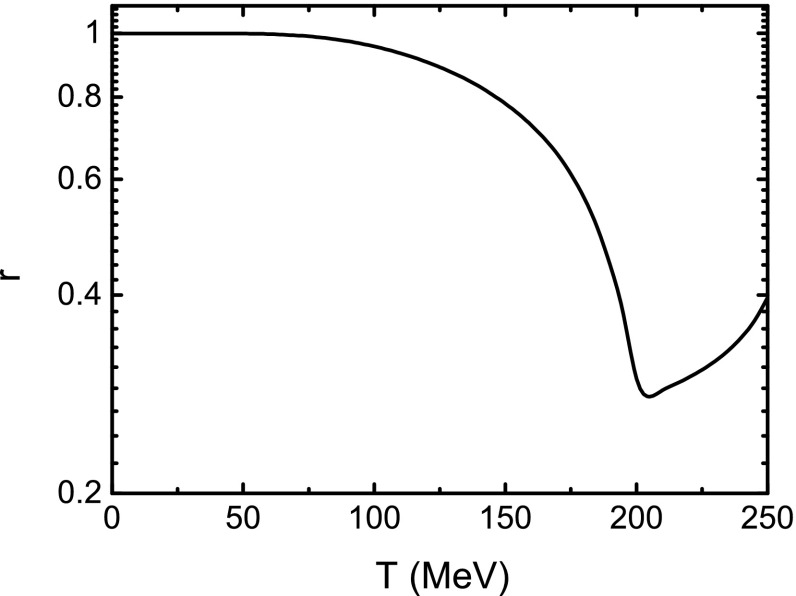
Calculated temperature dependence of the ratio *r* defined in Eq. (55) in the case of vanishing magnetic field

**Fig. 9:**
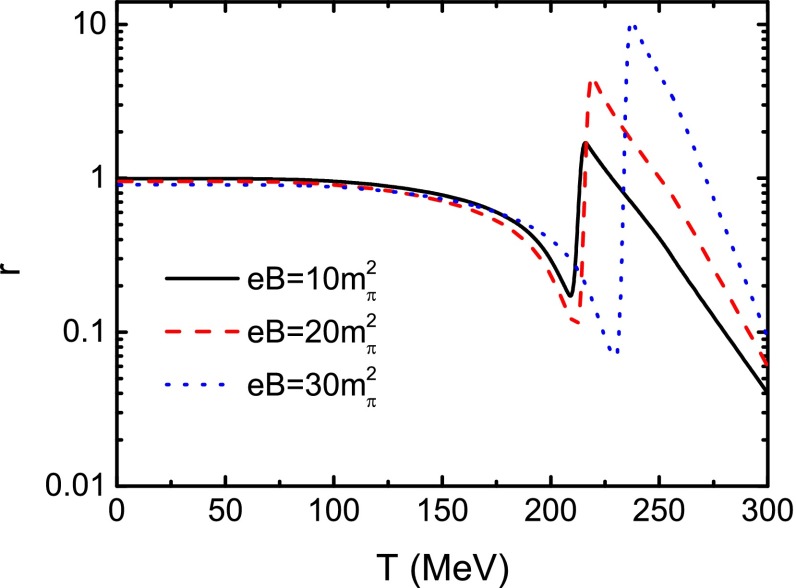
Calculated temperature dependence of the ratio *r* defined in Eq. (55) at several values of magnetic field strength *eB*

We show the calculated result of the ratio as a function of temperature without magnetic field in Fig. 8. It shows obviously that, at low temperature, the ratio stays almost constant, 1, which is a demonstration of the DCSB represented by the GOR relation. However, when the temperature increases, it deviates significantly from 1, which means that the temperature damages the GOR relation drastically or, in other words, induces the dynamical chiral symmetry to be restored. Furthermore, we illustrate the dependence of the ratio on the temperature in the case of a nonzero magnetic field strength in Fig. 9. One can recognize easily from Fig. 9 that with different strengths of the magnetic field, the ratio does not distinctly deviate from 1 either if the temperature is lower than the critical one. Once the temperature reaches up to around the critical value, *r* fluctuates seriously and both the temperature for the fluctuation to reach its first minimum and that for it to take its maximum increase with the ascension of the magnetic field strength, which implies that the fluctuation of the ratio *r* may be a signal for the chiral phase transition. The temperatures for the fluctuation to take its first minimum in several cases of the magnetic field are listed in Table 1. These characteristics indicate that the external magnetic field preserves the DCSB.

The same as for pions, we can get the masses of the neutral and the charged vector mesons $\rho ^0$ and $\rho ^\pm$ from the vector “polarization function” $\Pi _{\rho ^0}(p)$ and $\Pi _{\rho ^\pm }(p)$. For $\rho ^\pm$, we also make the point particle correction

56 $$\begin{aligned} M^2_{\rho ^\pm }(T,eB)=m^2_{\rho ^\pm }(T,eB)-eB. \end{aligned}$$

**Fig. 10:**
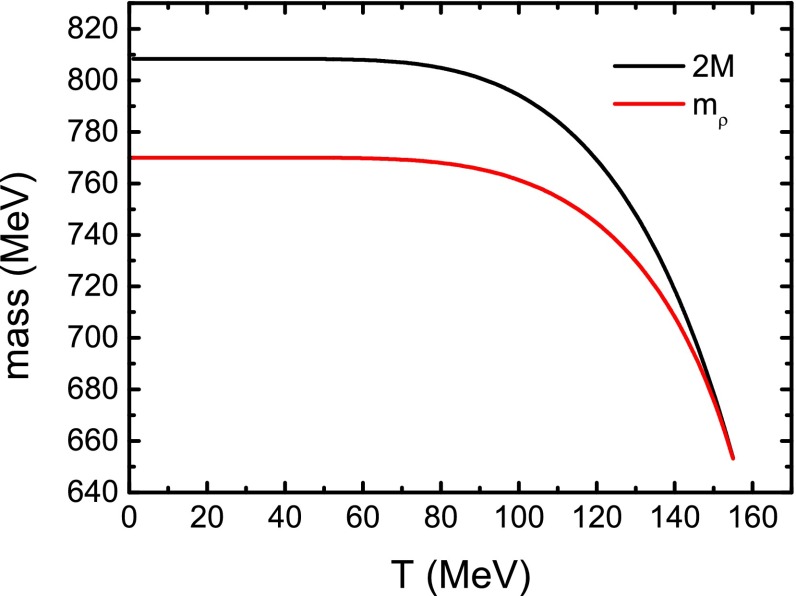
Calculated $\rho$ meson mass and twice the constituent quark mass as functions of temperature in the case of vanishing magnetic field

**Fig. 11:**
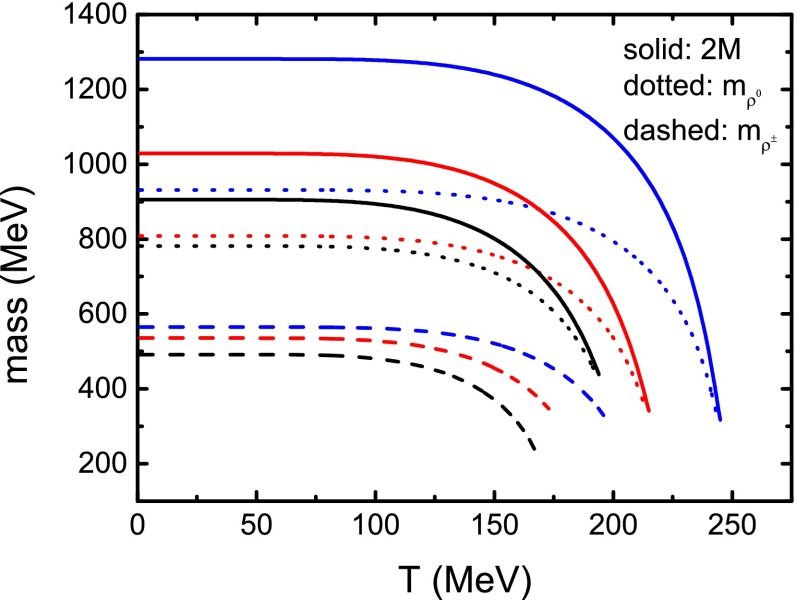
Calculated $\rho$ meson masses and twice the constituent quark mass as functions of temperature at several values of the magnetic field strength. The *black lines* stand for the results with $eB = 10 m_{\pi }^{2}$, *red* for those with $eB=20 m_{\pi }^{2}$, and *blue* for $eB = 30 m_{\pi }^{2}$

We consider at first the case of vanishing magnetic field where we cannot distinguish the charged $\rho ^\pm$ from the neutral $\rho ^{0}$, and illustrate the temperature dependence of the $\rho$ meson mass on the temperature in Fig. 10. The figure displays evidently that the $\rho$ meson mass decreases with temperature. When $T=155\;$MeV, the $\rho$ meson mass falls to the value of twice the constituent quark mass and there is no longer a solution for the $\rho$ meson mass at higher temperature. This phenomenon implies that at a critical temperature the $\rho$ meson gets disassociated, or melts to two quarks (more exactly, a quark and an antiquark), and in turn, there is no $\rho$ meson condensate at high temperature. In Fig. 11 we plot the results of both $\rho ^{0}$ and $\rho ^{\pm }$ meson masses and twice the constituent quark mass as functions of the temperature in the case of nonzero magnetic field strength. It shows that with finite magnetic field the $\rho ^{0}$ meson mass has the same behavior as that in the case without magnetic field, i.e., the $\rho ^{0}$ meson will melt at the critical temperature when its mass is equal to the twice of the constituent quark mass and the melting temperature increases with magnetic field strength. For the charged $\rho ^{\pm }$ in a magnetic field, the mass also decreases with temperature and remains smaller than the mass of the $\rho ^{0}$ meson. Similar to the behavior of $\rho ^0$, there are no longer solutions for the $\rho ^{\pm }$ mesons as the temperature gets higher than a critical value. It indicates that the $\rho ^{\pm }$ mesons also melt at high temperature, and the melting temperature is lower than that for the $\rho ^{0}$ meson in the same magnetic field. All the melting temperatures of $\rho ^{0}$ and $\rho ^{\pm }$ are also listed in Table 1 for comparison.

Inspecting Table 1, one can recognize that not only the pseudo-critical chiral symmetry restoration temperatures determined with different criteria but also the $\rho$ meson melting temperatures in the case without magnetic field are all smaller than those in nonzero magnetic field strength. The temperatures increase with strengthening the magnetic field. These features indicate that the external magnetic field can at least maintain the DCSB, so that there may exist magnetic catalysis in the region of the magnetic field strength we have considered. Comparing the melting temperatures of the neutral $\rho$ meson and the charged $\rho$ meson with the pseudo-critical temperature of the chiral phase crossover, one can find that the $\rho$ mesons will get melted as the DCSB is still quite strong if the magnetic field is not strong enough (for instance, $eB < 30 m_{\pi }^{2}$ for $\rho ^{\pm }$ and $eB < 10 m_{\pi }^{2}$ for $\rho ^{0}$), and in turn, there may not have been vector meson condensates in the QCD vacuum, which is consistent with the lattice QCD result [63] and the model calculation results [64, 65]. One may also infer that there exists magnetic inhibition for the vector hadrons.

## An alternative view

Considering the structure of the $\sigma$ meson discussed above, one may realize that it is the one having the quantum numbers of the vacuum, so that it plays a significant role in labeling the dynamical chiral symmetry restoration. However, it most likely does not correspond to the meson observed in QCD [114–121], since it has been well known that the $\sigma$ meson and the $\rho$ meson could be recognized as the resonant states of the $\pi$–$\pi$ scattering (see, e.g., Refs. [117–121]). In order to check the results we obtained in last section, we re-calculate the temperature and magnetic field strength dependence of the masses of the $\sigma$ meson and $\rho$ meson in the Roy equation [125] formalism of $\pi$–$\pi$ scattering [54, 117, 126–131]. To analyze the stability of the mesons in magnetic field, we also calculate the widths of the mesons’ mass poles.

It is well known that the significant inputs to determine the masses and their widths of the resonant states in $$\pi $$–$$\pi $$ scattering in the Roy equation scheme are the $$\pi $$–$$\pi $$ scattering lengths [117, 125, 127–131], which can be determined by the mass and the decay constant of the pion and the relation in the case of vanishing temperature and magnetic field has been well described in Ref. [126]. For convenience we outline the scheme and quote only the main formulas as follows. For the channel with isospin *I*, the scattering length $$a_{I} $$ can be determined by the scattering amplitudes $$T_{i}$$ ($$i=a,c,d,e$$ stands for the mode of scattering represented in terms of the Feynman diagrams shown in Fig. 1 of Ref. [126]), which can be fixed by the pion–quark–quark coupling constant, the “polarization functions” and so forth. After some calculation one has [126]

57 $$\begin{aligned} a_{0}= & {} \frac{7}{32\pi } \left( \frac{m_{\pi } }{f_{\pi } } \right) ^{2} \left[ 1 + \mathcal {O}( m_{\pi }^{2} ) \right] \, , \end{aligned}$$

58 $$\begin{aligned} a_{1}= & {} 0 \, , \end{aligned}$$

59 $$\begin{aligned} a_{2}= & {} \frac{-2}{32\pi } \left( \! \frac{m_{\pi } }{f_{\pi } } \! \right) ^{2} \left[ 1 \! - \! ( 1 \! - \! 5 z \! + \! \frac{9 }{4} z^{2} ) \frac{m_{\pi }^{2}}{4 M^{2}} \! + \! \mathcal {O}(m_{\pi }^{4}) \right] ,\nonumber \\ \end{aligned}$$

where $z = \frac{ \Lambda ^{4} M^{2}}{\pi ^{2} (\Lambda ^{2} + M^{2} )^{2} f_{\pi }^{2}}$, *M* is the constituent quark mass, $m_{\pi }$ is the pion mass, $f_{\pi }$ is the pion decay constant, and $\Lambda$ is the cutoff in the NJL model.

**Fig. 12:**
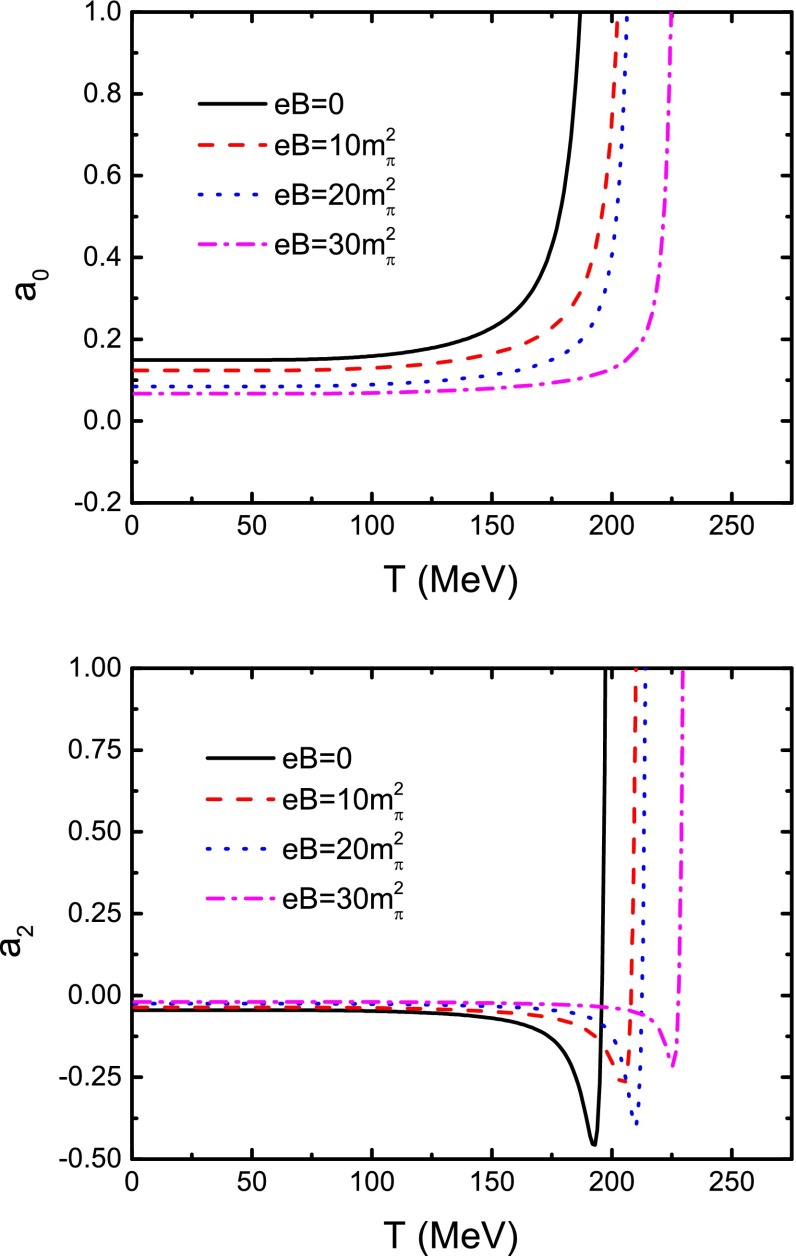
Calculated scattering lengths $$a_{0} $$ and $$a_{2} $$ as functions of temperature in cases of zero and several nonzero magnetic field strengths

It is apparent that the above relation can be extended to the case at finite temperature and finite magnetic field with only taking the *M*, $$m_{\pi }$$, $$f_{\pi } $$, and $$\Lambda $$ in the case of finite temperature and finite magnetic field as the inputs. With those obtained in the last section as the inputs we get the scattering lengths in the case of vanishing and nonzero magnetic field strength. The obtained results are shown in Fig. 12. The figure manifests evidently that, in both the cases of zero and nonzero magnetic field strength, $$a_{0} $$ and $$a_{2} $$ all remain correspondingly constant in low temperature region. With increasing magnetic field strength, the absolute value of the “constant” gets smaller. As the temperature increases to the pseudo-critical temperature denoted $$T_\mathrm{c}^{r}$$ in last section, both the $$a_{0} $$ and the $$a_{2} $$ diverge to positive infinity rapidly. Extending the discussion in Ref. [54], such divergences mean that the pion may get melted at the temperature. In addition, combining such a feature with the meaning of $$T_\mathrm{c}^{r}$$, we can infer that the dynamical chiral symmetry restoration and the quark deconfinement coincide with each other [20, 21].

**Fig. 13:**
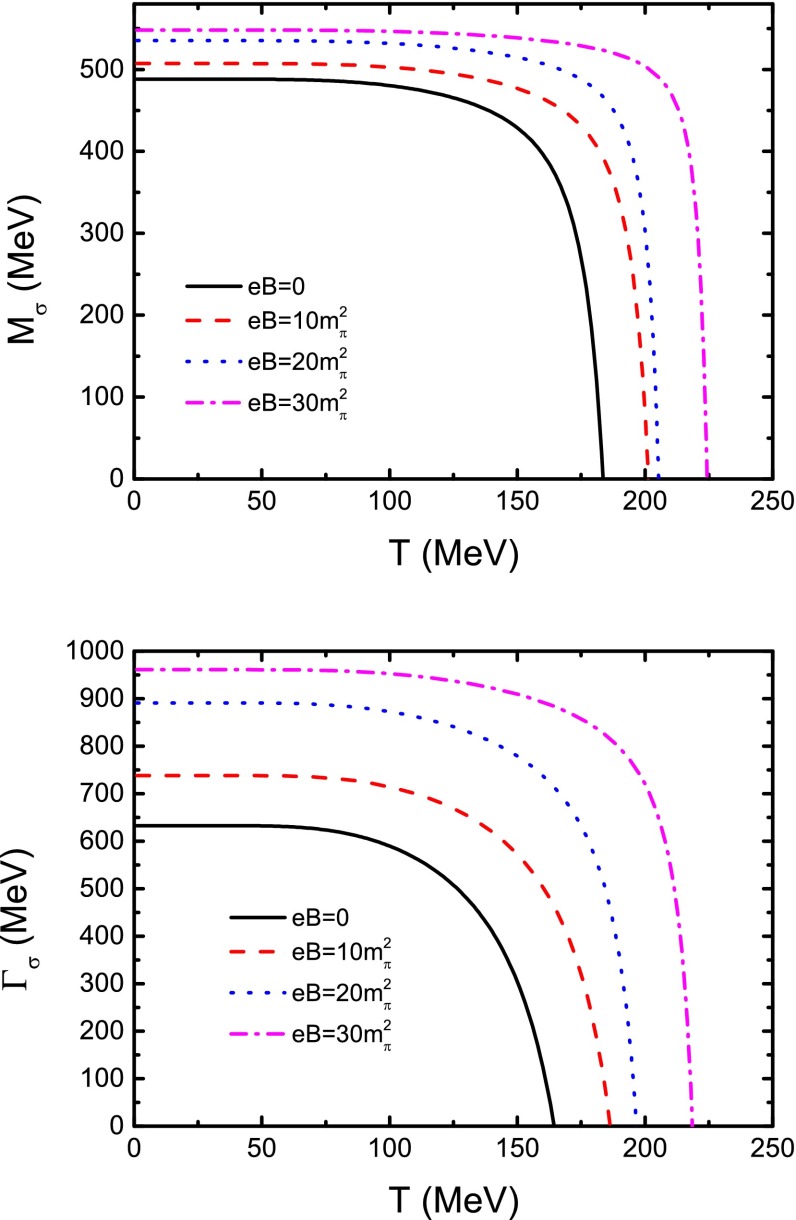
Calculated $\sigma$ meson mass and its width as functions of temperature in the cases of zero and several nonzero magnetic field strengths

Having the $\pi$–$\pi$ scattering lengths at hand, one can obtain the masses and their widths of the $\sigma$ and $\rho$ mesons in the scheme of the Roy equation [117, 125, 127–131]. Following the method of Ref. [130], we can fix the mass and width of the $\sigma$ meson as

60 $$\begin{aligned} m_{\sigma } = m_{0} + m_{1} \Delta a_{0} + m_{2} \Delta a_{2}, \end{aligned}$$

where

61 $$\begin{aligned} \Delta a_{0}= & {} (a_{0} - 0.22)/0.005\, , \end{aligned}$$

62 $$\begin{aligned} \Delta a_{2}= & {} (a_{2} +0.0444)/0.001 \, , \end{aligned}$$

with $m_{0} = 441 - i272$ MeV, $m_{1} = -2.4 + i3.8$ MeV, and $m_{2} = 0.8 - i4.0$ MeV. We can then have the temperature and magnetic field strength dependence of the $\sigma$ meson mass and width when the results illustrated in Fig. 12 are taken as the inputs. The obtained results of the temperature dependence of the mass and the width at zero and several nonzero magnetic field strengths are shown in Fig. 13. It shows obviously that, at zero temperature and zero magnetic field strength, $\sigma$ meson mass $m_{\sigma } = 488\;$MeV and its width $\Gamma _{\sigma } = 633\;$MeV. They all agree very well with the data given in PDG [119, 120] and Refs. [121, 130, 131]. We can also see from the figure that the variation behavior of the mass in the low temperature region is qualitatively consistent with the result we obtained by analyzing the internal quark–antiquark structure described in the last section except for that there exists a roughly factor 2 difference between the $m_{\sigma } (T\!=\!0, eB\!=\!0)$. The feature for the mass to decrease to 0 but not increase at high temperature is due to the divergence of the scattering length. Moreover, the decreasing characteristic of the width with respect to the temperature indicates that such a scalar meson may not melt at high temperature, which is consistent with the lattice QCD result for heavy scalar mesons [132, 133]. Figure 13 also makes manifest distinctly that the $\sigma$ meson mass increases with increasing magnetic field strength in the low temperature region, which is consistent with the result we obtained in the last section. Meanwhile the width of the $\sigma$ meson mass pole increases with the magnetic field strengths.

**Fig. 14:**
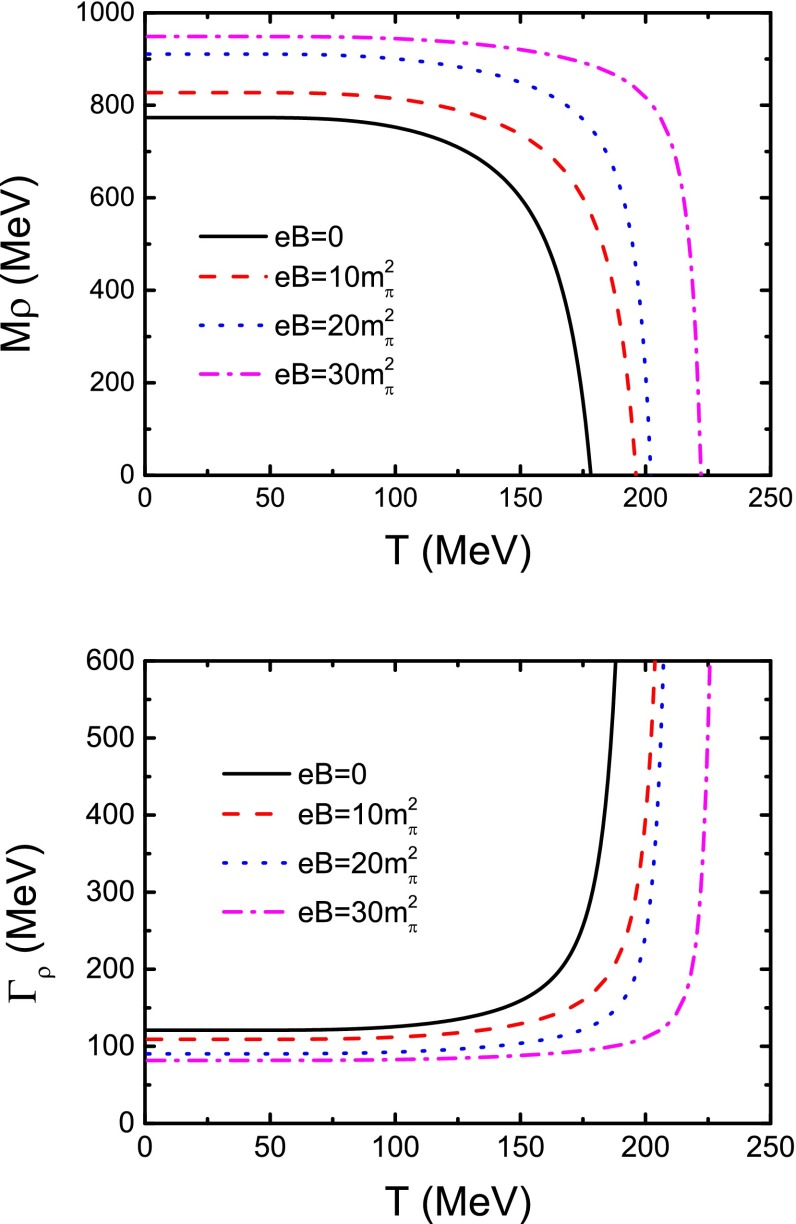
Calculated $\rho$ meson mass and its width as functions of temperature in the cases of zero and several nonzero magnetic field strengths

In a similar way, we can also fit the $\rho$ meson mass and width in view of $\pi$–$\pi$ scattering in the form

63 $$\begin{aligned} m_{\rho } = m_{0,\rho } + m_{1,\rho } \Delta a_{0} + m_{2,\rho } \Delta a_{2} \, , \end{aligned}$$

with the $\Delta a_{0}$ and $\Delta a_{2}$ being the same as Eqs. (61), (62), respectively, and parameters $m_{0,\rho } =715.5-i73.5\,$MeV, $m_{1,\rho } = -3.9 - i0.9\,$MeV, and $m_{2,\rho } = 4.5 + i0.2\,$MeV.

**Table 2:** Calculated critical temperature for $\rho$ meson mass to be zero ($T_\mathrm{c}^{\rho }$), the $\rho$ meson disassociation temperature ($T_\mathrm{da}^{\rho }$) and comparison with the melting temperature ($T_\mathrm{m}^{\rho }$) obtained in the last section (all the temperatures are in units of MeV and the *eB* in $m_{\pi }^{2}$ at zero temperature and zero magnetic field)

*eB*	0	10	20	30
$T_\mathrm{c}^{\rho }$	177	196	202	222
$T_\mathrm{da}^{\rho }$	187	204	207	226
$T_\mathrm{m}^{{\rho }^{0}}$	155	194	215	245

The obtained results of the temperature dependence of the $\rho$ meson mass and width at zero and several nonzero magnetic field strengths are shown in Fig. 14. It is apparent that the $\rho$ meson mass $774\,$MeV and width $121\,$MeV at zero temperature and zero magnetic field strength agree with the experimental data quoted in PDG excellently. Meanwhile, in the low temperature region, the $\rho$ meson mass almost maintains a constant value, and it decreases to zero quite rapidly as the temperature gets close to the critical value $T_\mathrm{c}^{\rho }$. With the increasing of the magnetic field strength, both the constant value and the critical temperature ascend (some values of the $T_\mathrm{c}^{\rho }$ are listed in Table 2). Such a feature is exactly the same as we obtained in the last section. Furthermore our calculations make manifest that at any finite magnetic field strength, the width of the $\rho$ meson mass pole increases with the increasing of temperature; at a certain temperature, it decreases as the magnetic field gets stronger. Especially the width diverges at a certain critical temperature which increases as the magnetic field strength becomes larger. The divergence of the $\rho$ meson mass width means that the life-time of the $\rho$ meson becomes zero, so that the $\rho$ meson will disassociate at the critical temperature. The obtained divergence temperature or the disassociation temperature $T_\mathrm{da}^{\rho }$ is listed in Table 2. For comparison we re-quote the temperature for the $\rho _{0}$ meson to melt, $T_\mathrm{m}^{\rho _{0} }$, in Table 2. The figure and the table show obviously that the properties of the $\rho$ meson in view of the resonant state of pion–pion scattering are just the same as those obtained with analyzing the internal quark–antiquark structure of the mesons in the last section.

## Summary and remarks

In this paper, we have calculated some properties of the scalar meson $\sigma$, pseudoscalar meson $\pi ^{0,\pm }$, and the vector meson $\rho ^{0,\pm }$ at finite temperature and finite magnetic field in two distinct schemes in the NJL model. One is the conventional scheme, which takes the mesons as quark and antiquark bound states. Another is one that regards the $\sigma$ and $\rho$ mesons as the pion–pion scattering resonant states.

To calculate the masses of the mesons sophisticatedly in the NJL model, we extend the $\Phi$-derivable method to a finite magnetic field at first. Our calculation results make manifest that the mass of the $\sigma$ meson in magnetic field keeps the same behavior as that in the case of zero magnetic field, but with increasing magnetic field, the temperature dependence of the $\sigma$ meson mass becomes weaker. For the pseudoscalar meson $\pi$, the behavior becomes a little complicated. In a finite magnetic field, the neutral $\pi ^{0}$ and the charged $\pi ^{\pm }$ separate from each other, but they have similar dependence behaviors on the temperature, except for a slight quantitative difference. When the temperature is lower than the critical value for nonzero magnetic field, the $\pi ^{0}$ mass keeps almost a constant value. Once the temperature reaches the critical value, the $\pi ^{0}$ mass increases abruptly with the increase of the temperature, and it becomes degenerate with the $\sigma$ meson mass. However, the degeneracy is not precise because of the magnetic catalysis and the finite current quark mass effect. The charged $\pi ^{\pm }$ mass increases with the magnetic field, no matter if the temperature is lower or higher than the critical value. We also find that the critical temperature obtained from the $\pi$ mass is overall a little higher than that gained by analyzing the properties of the quark. Such a feature, that different criteria give distinct critical temperatures, implies that the chiral phase transition at finite temperature and finite magnetic field is a crossover. For the vector meson, we also distinguish the neutral $\rho ^0$ from the charged $\rho ^{\pm }$ in our calculation. The obtained results display the masses of not only the neutral but also the charged particles increase with the strengthening of the magnetic field at low temperature. At a certain magnetic field, the masses decrease generally with the increasing of temperature. When the temperature increases to the critical value, both the $\rho ^{0}$ and the $\rho ^{\pm }$ mass solutions disappear, which implies that the vector mesons will melt. The melting temperature increases with the ascension of the magnetic field, and the $\rho ^{0}$ melting temperature is slightly higher than that for $\rho ^{\pm }$. Because the $\rho$ meson will melt at high temperature, there may not exist a $\rho$ meson condensate in the QCD vacuum.

We have also calculated the temperature and the magnetic field strength dependence of the neutral pion decay constant and checked the GOR relation in the case of finite temperature and finite magnetic field. Our calculation results of the decay constant agree very well with the previous one. Meanwhile, we find that the temperature influences the GOR relation more than the magnetic field and the fluctuation of the ratio $r=\frac{f_{\pi }^{2} m_{\pi }^{2}}{-m_{0} \langle \overline{q}q\rangle }$ can be a signal for the chiral phase transition. Such an aspect shows again that the magnetic field preserves the DCSB.

To calculate and analyze the properties of the $\sigma$ and $\rho$ mesons in the case of vanishing and nonzero magnetic field strength in the pion–pion scattering scenario, we take the formalism of the Roy equation by extending, for the first time, the calculation of the $\pi$–$\pi$ scattering lengths to finite magnetic field. The masses and their widths at zero temperature and zero magnetic field strength we obtained excellently agree with the experimental data. Our calculated results of the temperature and magnetic field strength dependence of the scattering lengths ($a^{0}$ and $a^{2}$) and the mass widths indicate that the $\pi$ meson and the $\rho$ meson will get disassociated at high temperature and strong magnetic field, and the disassociation temperature of each kind of the mesons is almost the same as the corresponding melting temperature obtained by analyzing the internal quark structure. Meanwhile increasing the magnetic field strength retards the disassociation. These features confirm that there does not exist a (charged) vector meson condensate in the QCD vacuum at finite magnetic field. The scalar meson $\sigma$ will not disassociate, which agrees with what a lattice QCD calculation on heavy flavor mesons makes manifest.

All the obtained variation behaviors of the mesons’ properties with respect to the temperature and the magnetic field strength provide further evidence that the external magnetic field enlarges the dynamical chiral symmetry breaking area, i.e., there exists a magnetic catalysis. However, it does not mean that we have reached the end, since we have not taken into account explicitly the magnetic inhibition [87], the sphaleron [90], and other effects. Furthermore the NJL model is only a contact interaction approximation of the strong interaction, which neglects the contributions of the complicated quark–gluon interaction vertex and the dressed gluon propagator. Extending the result obtained in the linear sigma model [134], one may infer that such a neglect should be the origin of the magnetic catalysis in the models. In addition, we have not taken into account the temperature and magnetic field strength dependence of the cutoff in the calculations, either. Investigations on the meson properties with more sophisticated approaches (e.g., the Dyson–Schwinger equation approach, incorporating explicitly the magnetic field dependence of the quark–gluon interaction vertex and the gluon propagator, and so on) are necessary. On the other hand, the practical situation may, in fact, be more complicated, for instance, the effect of the magnetic field on the phase transition may depend on the field strength non-monotonically.
